# Strong indication of an extinction‐based saturation of the flora on the Pacific Robinson Crusoe Islands

**DOI:** 10.1002/ece3.3882

**Published:** 2018-02-01

**Authors:** Josef Greimler, Christian H. Schulze, Patricio López Sepúlveda, Patricio Novoa, Alejandro Gatica, Karl Reiter, Johannes Wessely, Carlos Baeza, Patricio Peñailillo, Eduardo Ruiz, Tod Stuessy

**Affiliations:** ^1^ Department of Botany and Biodiversity Research University of Vienna Vienna Austria; ^2^ Departamento de Botánica Universidad de Concepción Concepción Chile; ^3^ Jardin Botánico Corporación Nacional Forestal Viña del Mar Chile; ^4^ Instituto de Ecología y Biodiversidad (IEB) Universidad de Chile Ñuñoa Chile; ^5^ Departamento de Biología Vegetal y Biotecnología Universidad de Talca Talca Chile; ^6^ Herbarium, Museum of Biological Diversity The Ohio State University Columbus OH USA

**Keywords:** 100 year comparison, alien plants, extinction‐based saturation, island flora, Robinson Crusoe Islands, vegetation change

## Abstract

Oceanic islands are vulnerable ecosystems and their flora has been under pressure since the arrival of the first humans. Human activities and both deliberately and inadvertently introduced biota have had and continue to have a severe impact on island endemic plants. The number of alien plants has increased nearly linearly on many islands, perhaps resulting in extinction‐based saturation of island floras. Here, we provide evidence for such a scenario in Alejandro Selkirk, Robinson Crusoe Islands (Archipelago Juan Fernández, Chile). We compared species richness and species composition of historical vegetation samples from 1917 with recent ones from 2011. Changes in species’ relative occurrence frequency were related to their taxonomic affiliation, dispersal mode, distribution status, and humidity and temperature preferences. While total species richness of vascular plants remained relatively similar, species composition changed significantly. Plants endemic to the Robinson Crusoe Islands declined, exotic species increased substantially within the period of ca. 100 years. Further, the relative occurrence frequency of plants with preferences for very warm and humid climate decreased, while the opposite was found for plants preferring drier and colder environments. Potential drivers responsible for this dramatic shift in the vegetation within only one century might have been the large goat population affecting especially small populations of endemic plants and climatic changes. Taking into account a substantial extinction debt, we expect further shifts in the vegetation of this small oceanic island toward alien plants. This would have significant negative consequences on global biodiversity, considering that island floras contribute substantially to global plant species richness due to their high proportion of endemics.

## 1 INTRODUCTION

Oceanic islands and their organisms provide unique natural laboratories to study plant evolution. In general, island floras are strongly shaped by factors such as isolation, geomorphology, age, and the regional climate causing a high stochasticity of colonization patterns (Mueller‐Dombois & Fosberg, 1998; Thornton, 2007). As a result of founder and drift effects, and various forms of selection, especially driven by the ecological release from herbivores (Whittaker & Fernández‐Palacios, 2007), particularly remote islands harbor many endemic plants. However, the several waves of prehistoric and historic human colonization of oceanic islands combined with the introduction of alien biota including formerly absent herbivores have resulted in a severe menace for those plants. Besides the impact from alien organisms, climate change is considered a severe issue for island biota although climate change per se may not be a main driver of biodiversity loss but rather in combination with existing stress (Heywood, 2011). However, climate change may have a more severe effect on small islands given that some features of the “island syndrome” such as longevity, change in breeding system, and loss of dispersibility limit the possibility to escape the changing climate via migration (Bramwell, 2011). Although extinctions of native (incl. endemic) island plants are comparatively rare compared to animals (Sax & Gaines, 2008), there are well‐documented cases of extinction in many angiosperm trees and palms across the Pacific islands (Prebble & Dowe, 2008) besides the overall gloomy scenario that up to 10% of the estimated 70,000 insular endemic plant species might be highly threatened and up to 4% of these species are even facing a high extinction risk (Caujape‐Castells et al., 2010).

Unfortunately, forecasts of future extinctions on islands are highly limited due to our relatively poor understanding of factors restricting the number of species that can inhabit islands (Sax & Gaines, 2008). However, it appears very likely that any island has its characteristic saturation point, at which its carrying capacity in terms of species richness will be reached. When a successful colonization of an island by new species at the island's saturation point results in the local extinctions of a similar number of previously established species, this “extinction‐based saturation” is consistent with the classical concept of the Island Biogeography Theory (MacArthur & Wilson, 1967; Sax & Gaines, 2008). When already present plants are able to inhibit the colonization of islands by new invaders, a “colonization‐based saturation” might be prevalent (Sax & Gaines, 2008). From a conservational perspective, both scenarios would have different consequences for the extinction risk of native plants. Once the saturation point will be reached, extinction‐based saturation would result in a shift in species composition toward a higher proportion of invasive species and an increased extinction of native species. Colonization‐based saturation predicts that exotic species will not further be able to establish as soon as the saturation point will be reached, while the displacement of native species will be very unlikely. In this study, we use long‐term data from the Robinson Crusoe islands (Archipelago Juan Fernández) to evaluate if one of these two competing concepts is able to explain the recent shifts in plant species composition.

Repeated floristic surveys provide essentially the knowledge on decline and increase in endemics and aliens, respectively. As a rule, those surveys give overall information on a species’ occurrence frequency in general terms such as “rare, common, and frequent.” In addition, there has been an enhanced endeavor to identify and monitor the performance of novel alien species. Precise data, however, are nearly always missing for those past times, when the first complete surveys were done. The Robinson Crusoe islands are a lucky exception in this respect. The Swedish botanist Carl Skottsberg collected quantitative data on the vegetation of the archipelago in 1916 and 1917, published not until 1953 (Skottsberg, 1953). Vegetation relevés such as those applied by Skottsberg contain all species of a given plot and given a high number of those relevés provide a more precise picture on the vegetation and of a species’ abundance than floristic records. He noticed many alien plants (Skottsberg, 1922, 1953), and more recent floristic surveys have confirmed a steady increase in them (Swenson, Stuessy, Baeza, & Crawford, 1997; Danton, Perrier, & Reyes, 2006). Using recent (Greimler, Lopez, Stuessy, & Dirnböck, 2002) and the historical (Skottsberg, 1953) vegetation data of Robinson Crusoe Island, we could demonstrate a significant increase in the frequency of alien species (Greimler, Stuessy, Swenson, Baeza, & Mathei, 2002) contrasted by a notable decline in the endemics. Based on this, we provided a predictive modeling that revealed an unfavorable scenario for the endemic plants (Dirnböck, Greimler, Lopez, & Stuessy, 2003).

Here, we focus on the second large island Alejandro Selkirk of the archipelago that provides another rare case, where quantitative vegetation data collected nearly 100 years ago in 1917 (Skottsberg, 1953) are available and can be compared to recent data collected in 2011 (Greimler et al., 2013). In detail, we tested for shifts (i) in the proportions among endemic, native nonendemic, and alien species; (ii) in the species’ preferences for moisture and temperature; and (iii) in pollination and dispersal mode of species.

## 2 METHODS

### 2.1 Study area

**Figure 1:**
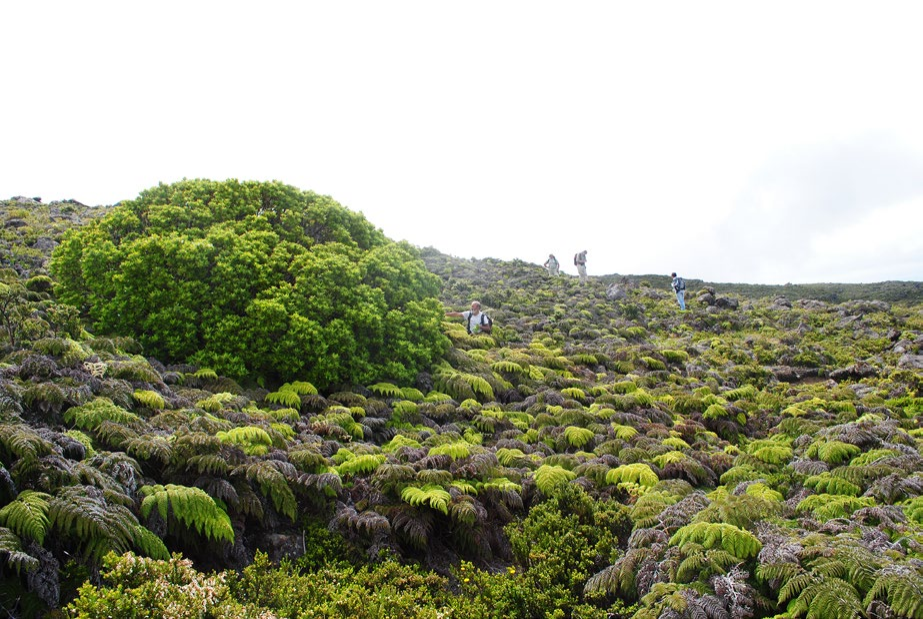
Endemic tree *Drimys confertifolia* within a matrix of the tall fern *Lophosoria quadripinnata* with a few shrubs and herbs. This tall fern community alternating with an alien‐rich grassland is the dominant vegetation pattern in the central and northern part of the island Alejandro Selkirk

The Robinson Crusoe Islands (Archipelago Juan Fernández) are situated between 640 and 850 km west of continental Chile in the Pacific Ocean. The archipelago comprises two large islands, Robinson Crusoe (Masatierra), 33°36′–33°40′S and 78°46′–78°56′W, and the more remote Alejandro Selkirk (Masafuera), 33°43′–33°49′S and 80°45′–80°50′W (see Figure 1 for the dominant fern assemblage there), plus a smaller island, Santa Clara, near Robinson Crusoe. The archipelago is a Chilean national park and Biosphere Reserve.

### 2.2 Vegetation data

Taxa recorded by Skottsberg (1953) were adjusted accordingly. We treated the natural habitats of the whole island as a single meta‐assemblage and compared over several vegetation types between the two sets of vegetation relevés. This has remained the only feasible approach as we were not able to perform an exact site resampling in most cases due to imprecise site data for the old collections (Table S1), which allowed only a very rough estimate where these could have been (more on this in the Supporting Information). In Table S2, the locations of our resurvey samples are given. Overall these resurvey data provide a mixture of resampling on quasi‐permanent plots and nontraceable plots according to Kapfer et al. (2016). By including a high number of relevés (inside and outside the circles in Figure S1) from only those plant assemblages that are well represented in Skottsberg's data, we assume to have reduced sampling bias and pseudoturnover as much as possible. The plant assemblages were not evenly represented in both data sets. Therefore, we excluded all grassland relevés from both datasets as those are extremely underrepresented in Skottsberg's relevés (Skottsberg, 1953) (only two compared to 34 in Greimler et al., 2013). We also excluded all relevés of the plant assemblages on the coastal rocks as most of them were taken near the village. Additionally, the historical data had to be cleared from mosses, hepatics, lichens, which were not included in our relevés. Abundance/dominance data was recorded as simple presences.

The resulting data set then comprised 79 relevés (27 historical and 52 recent ones). The species with their attributes are listed in Table S3. The historical sampling sites are indicated by the green circles A–O in Figure S1) with each circle containing between one and three relevés of Skottsberg (s + number) and one to several recent relevés. The recent relevés (inside and outside these circles) are indicated by yellow dots. Information on the sampling sites of the included relevés is given in Tables S1 and S2. Taxonomy and nomenclature of the taxa followed Danton et al. (2006) and Marticorena, Stuessy, and Baeza (1998).

### 2.3 Explaining variables

All explanatory variables were categorical. The taxonomic affiliation of species was classified as monocots, dicots, or ferns, additionally with family and genus for a nested set of random effects. Species’ distribution status was classified as being native nonendemic, endemic (to the Robinson Crusoe Islands), or alien according to the most recent catalogue (Danton et al., 2006). Humidity and temperature preferences were derived from expert knowledge using the median of each vector of indicator values on a threefold scale assigned to each species by six independent experts (Tables S4 and S5). Moisture preferences were classified as “dry” (including plant species with the values 1.0 and 1.5 to achieve group size sufficient for statistical analysis), “indifferent” (2.0), “humid” (2.5), and “very humid” (3.0). As the m(temp) indicator value 1 appeared only once (for the grass *Chaetotropis imberbis*), it was included in the group of plants which achieved a median score of 1.5. Temperature preferences of plant species were then classified as being “very warm” (equals values of 1.0 and 1.5), “warm” (2.0), “indifferent” (2.5), and “cold” (3.0). Information on pollination and dispersal mode of species was extracted from Bernardello, Anderson, Stuessy, and Crawford (2002, 2006). The mixed dispersal category zoo/anemochorous was treated as a separate category. All data on the explanatory variables are given in Table S3 immediately after the species names.

### 2.4 Climate data

There are no data available for Alejandro Selkirk Island. In addition to the data provided by Falvey and Garreaud (2009) and Gobierno de Chile (2016), we checked the available data (Instituto Central Meteorológico y Geofísico de Chile, 1922; Oficina Meteorológica de Chile, 1934; Dirección Meteorológica de Chile, 1977) covering more or less the last century of the neighboring Robinson Crusoe Island and the nearby meteorological station of Valparaiso on the continent. While we could not find evidence for a change in annual precipitation on Robinson Crusoe Island over the whole century, we found a solid trend toward lower precipitation since the 1970s (Figure S2A). The data of Valparaiso are inconclusive because of too many missing values. The temperature trends (Figure S2B,C) show a slight decrease in the mean temperature on Robinson Crusoe island (1935–2014: <0.5°C) and a notable decrease in Valparaiso (1914–1998: >1°C).

### 2.5 Data analysis

To compare species richness of both survey periods, we calculated species accumulation curves (±95% CI) for both periods and extrapolated them to a total of 120 samples (=plots) using EstimateS vers. 9.1.0 (Colwell, 2013). To compare species composition between plots and mapping periods, Soerensen similarities were calculated for all possible plot × mapping year pairings with the software Primer 5.2 (Clarke & Gorley, 2001). Nonmetric multidimensional scaling was applied to visualize similarity relationships. A *stress* value (a measure of poorness‐of‐fit) of <0.20 was considered to adequately represent species composition relationships (Clarke, 1993). To test for a difference in species composition of plots between mapping years a one‐way ANOSIM with 999 random permutations was calculated on the similarity matrix with the software Primer 5.2 (Clarke & Gorley, 2001). Simple chi‐square tests were used to test for differences in the distribution of native nonendemic, endemic, and alien species, separately for all taxa, monocots, dicots, and ferns. A linear mixed effect model calculated with R (version 3.4.2, package “afex”) was used to evaluate effects of taxonomic affiliation, species’ distribution status, humidity and temperature preferences, dispersal and pollination mode on species’ occurrence frequency. Species with missing values were excluded from model estimates. We used a nested grouping structure with genus in family in taxonomic group. The least square means predicted by this model were used to visualize the effects of species’ distribution status, humidity, and temperature preferences. Occurrence frequency was quantified as percentage of plots from which a species was recorded. Changes in occurrence frequency of species were quantified as differences in occurrence frequency between both survey periods (Table S3).

## 3 RESULTS

**Figure 2:**
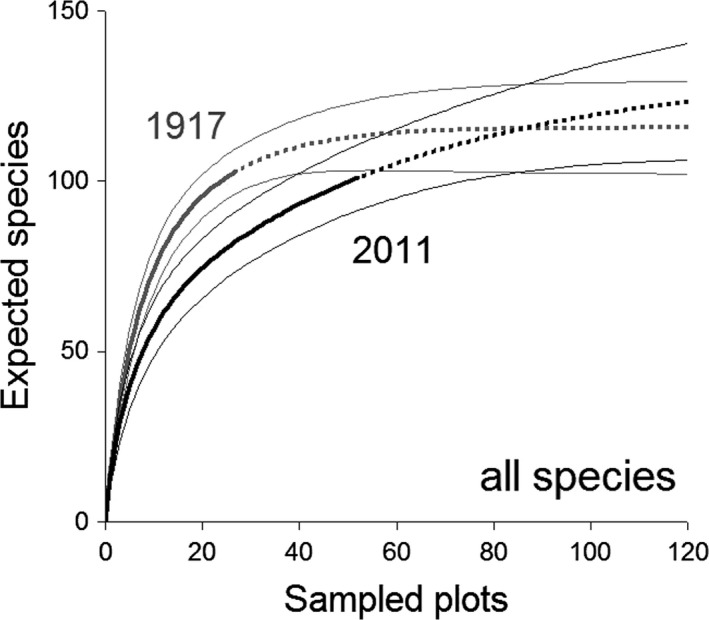
Plant species richness in both mapping periods. Species richness accumulation curves (±95% CI; dotted lines indicate extrapolated parts of the curves) calculated for both mapping periods 1917 and 2011 indicate similar species richness

**Figure 3:**
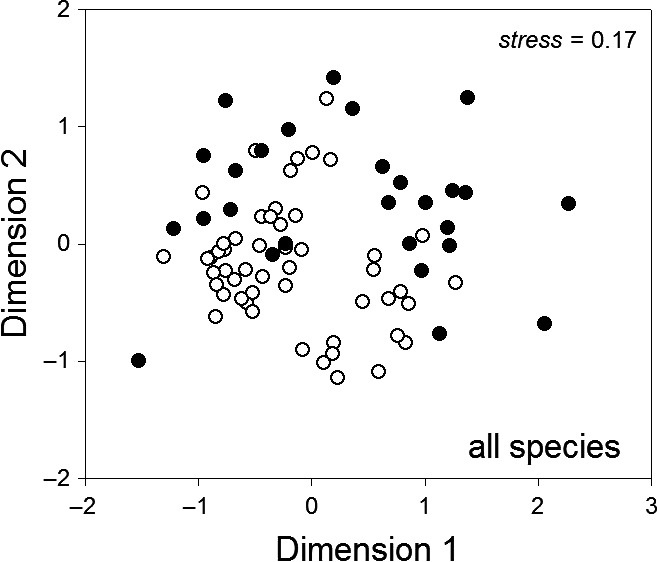
Similarity relationships in species composition between both mapping periods. To visualize similarity relationships between plant assemblages of individual plots and the two mapping periods 1917 (filled circles) and 2011 (open circles), nonmetric multidimensional scaling based on Soerensen similarities was applied. Plant species composition differed significantly between both years (one‐way ANOSIM: Global *R *=* *.331, *p *=* *.001)

**Figure 4:**
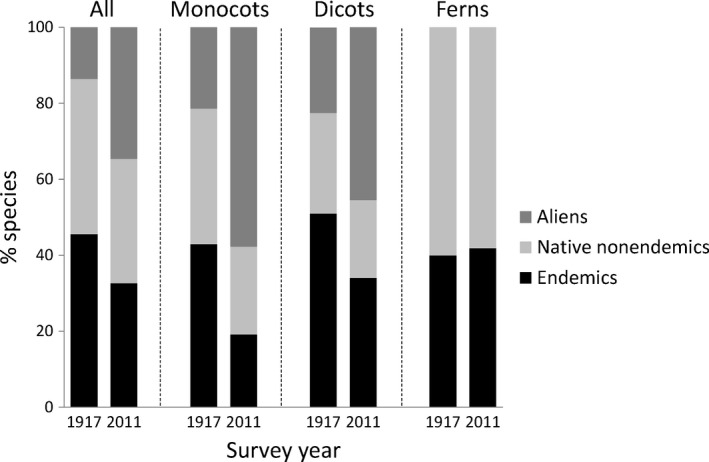
Change in the relative richness of species with different distribution status between both mapping years. Proportion of endemic, native nonendemic, and alien species recorded in 1917 and 2011, respectively, shown for all plants and separately for monocots, dicots, and ferns (no alien species recorded in latter group)

Nearly identical total plant species richness was recorded in 1917 (103 species) and 2011 (101 species). Very similar species richness is also indicated by comparing the species accumulation curves for both periods (Figure 2). However, species composition differed between 1917 and 2011 (Figure 3) due to a dramatic shift toward a higher proportion of alien species (chi‐square test: χ^2^ = 12.51, *df* = 2, *p *=* *.0019; Figure 4). Although this shift was also visible for monocots and dicots, differences did not achieve a significant level (monocots χ^2^ = 5.04, *df* = 2, *p *=* *.0807; dicots (χ^2^ = 5.59, *df* = 2, *p *=* *.0612). So far, no alien fern species were recorded and the proportion of native nonendemic and endemic species did not change significantly between both survey periods (χ^2^ = .03, *df* = 1, *p *=* *.8693; Figure 4).

**Figure 5:**
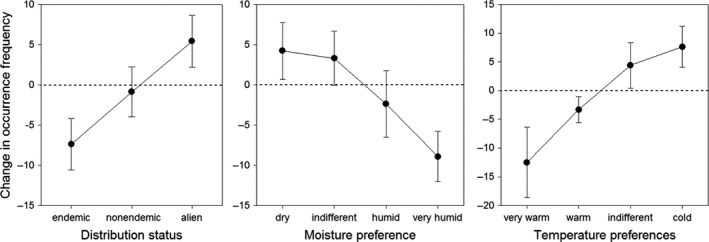
Trait‐related changes in occurrence frequency of plant species. Mean changes (±*SE*) in occurrence frequency (least square means predicted by the mixed effect model evaluating effects of various traits) of species with different distribution status, and humidity and temperature preferences. While alien species and species with a preference for dry and cold climate increased, endemic species and species favouring a more humid and very warm climate declined between 1917 and 2011

A linear mixed model testing for effects of species’ distribution status, moisture and temperature preferences, dispersal and pollination mode indicates clear effects of distribution status and climatic preferences on changes in species’ occurrence frequency (Table 1). While occurrence frequency of alien and endemic species increased and decreased, respectively, occurrence frequency of native nonendemic species remained similar. Occurrence frequency of species with preferences for dry and cold environments increased; species with preferences for humid and warm environments showed an opposite pattern (Figure 5).

**Table 1:** Effects of species’ traits on changes in occurrence frequency of plant species

Fixed effects	*df*	den *df*	*F*	Pr (>*F*)
Status	**3**	**108.0331**	**7.7384**	**0.000723**
Moisture preference	**3**	**86.19674**	**4.4281**	**0.006059**
Temperature preference	**3**	**87.28332**	**4.1588**	**0.008379**
Pollination mode	3	0.78515	0.0253	0.991355
Dispersal mode	4	3.88461	0.8382	0.567336

The Mixed Modell evaluating effects of species’ traits on changes in occurrence frequency was calculated with genus nested in family nested in taxonomic group. Significant effects are printed in bold.

## 4 DISCUSSION

Comparing the quantitative vegetation surveys of 1917 (Skottsberg, 1953) and 2011 (Greimler et al., 2013) revealed a significant decline in endemic species on Alejandro Selkirk. A few of those taxa present in 1917 are now considered extinct (Danton et al., 2006), others have become too rare to be recorded by our survey. For instance, each of the three species of the endemic genus *Dendroseris* has been recorded more than once in 1917 but none of them in 2011. Their populations have obviously become very small with the extreme case of only one or two known individuals of *Dendroseris gigantea* and *D. macrophylla* at present (Danton et al., 2006; Ricci, 2006). One hundred years ago, their incidence was estimated “scattered” and “not uncommon,” respectively (Skottsberg, 1953). Given the small population sizes of many endemic and some native plants on this island, we suspect a substantial “extinction debt” (Sax & Gaines, 2008) that will be paid in the near future. These results provide strong evidence for an extinction‐based saturation currently shaping the island flora of Alejandro Selkirk.

Introduced goats have had and continue to have a negative effect on the flora of the island. Some highly endangered endemic plants are included in their diet (Cuevas & van Leersum, 2001). The detrimental effect of goats on the native flora of oceanic islands is well documented for several Pacific (Merlin & Juvik, 1992) and Atlantic Islands (Gangoso, Donazar, Scholz, Palacios, & Hiraldo, 2006; Alves, da Silva, & Aguirre‐Munoz, 2011). The animals have been introduced on Alejandro Selkirk in the late 16th century (Jaksic, 1998). They may have increased in number until the end of the 18th century when the island was used for some decades as a penal colony and when there was high human impact in general during the sealing and whaling period (1798–1808). At least since then, the goat population has suffered recurring impacts from hunting and population control measures, causing the population to fluctuate between a minimum of 500 and a maximum of 2,000–3,000 goats. Estimates from CONAF (Corporación Naciónal Forestál) park service for 2011 were 1,200–2,000 goats.

In contrast to the decline of endemic plants, alien plants appeared with significantly higher frequencies in the 2011 relevés. Two of the three most common aliens (*Anthoxanthum odoratum* and *Rumex acetosella*) were found at very low frequencies in the 1917 relevés, and one (*Hypochaeris radicata*) is entirely missing in all of Skottsberg's records (Skottsberg, 1922, 1953). Most likely *H. radicata* did arrive after 1917 on Alejandro Selkirk and rapidly did invade nearly all plant assemblages in less than 100 years. Goats may still have played a substantial role in this change. There is evidence from several Pacific islands that the presence of feral ungulates has facilitated alien plant invasion (Merlin & Juvik, 1992). Another reason for the rapid increase in alien species on oceanic islands globally may lie in their feature of having functional traits that tend to be missing in island floras providing them with the ability to colonize empty niches (Kueffer et al., 2010). Although alien plants do compete with the native flora, direct competition seems to be of minor importance in causing endemic plant extinctions (Sax & Gaines, 2008).

Endemic plants of Alejandro Selkirk are exposed to the combined effect of grazing pressure by feral goats and many human activities (e.g., logging, hunting, animal husbandry, gardening) both facilitating seed/ramet dispersal and establishment of alien plant populations and a steady increase in their propagule pressure. Besides those determinants, climate change may be another driver of vegetation change. The plant assemblages of 2011 indicate a slightly increased tolerance for drier conditions and for lower temperatures. From a general southern hemisphere temperature increase within the past century (Neukom et al., 2014), we initially assumed that Skottsberg's data (Skottsberg, 1953) were collected at the end of a more humid and cooler period. However, the regional temperature patterns contradict the global patterns with a cooling in the southeastern Pacific and along the Chilean coast (Falvey & Garreaud, 2009; Gobierno de Chile 2016) besides a reduced precipitation along the coast (Gobierno de Chile 2016).

The native and endemic flora of the island Alejandro Selkirk is endangered simply by the small size of the island because plant endemics of small oceanic islands are more often endangered by stochastic events than those of larger ones (Caujape‐Castells et al., 2010). Very pessimistic statements (Cuevas & van Leersum, 2001) assume that 75% of the endemic vascular plant flora of the archipelago is on the “verge of extinction.” Although this figure may be exaggerated, we assume that there will be a substantial “extinction debt” that will be paid in near future. The similar species richness per plot recorded for both survey periods indicates that the studied island flora may have reached its saturation point in the natural habitats of higher elevations and an extinction‐based saturation is currently operating. The reported negative consequences for the endemic plants may be additionally facilitated by a high human‐induced herbivorous predation which may keep the species richness permanently slightly below the saturation point, hence continuously providing opportunities for invasive plants to establish. Particularly, many of the species with small population size and unfavorable population structure will not be able to respond successfully (e.g., by adapting their reproductive strategies) to the changed environmental conditions and the occurrence of new competitors.

## AUTHOR CONTRIBUTIONS

J.G. designed the study, collected and arranged the data, and wrote the paper together with C.H.S., who provided the statistical design and analysis together with J.W.. The expert system was developed by J.G., P.L., P.N., C.B., P.P., and T.S. These authors and E.R. contributed to resolve the old synonyms. A.G. and P.N. contributed in data collection on climate and goat impact history; K.R. in data and map processing. All authors discussed the results and commented on the manuscript.
