# Supplementary material for: Strong indication of an extinction‐based saturation of the flora on the Pacific Robinson Crusoe Islands
Source: Ecol Evol. 2018 Feb 1;8(5):2527–33. doi: 10.1002/ece3.3882 (PMC5838048; doi:10.1002/ece3.3882)
Supplement: Supplementary file 1 [file ECE3-8-2527-s001.docx]

**SUPPORTING INFORMATION**

Strong indication of an extinction-based saturation of the flora on the Pacific Robinson Crusoe Islands

**Short title: Extinction-based saturation of an island flora**

Josef Greimler, Christian H. Schulze, Patricio López, Patricio Novoa, Alejandro Gatica, Karl Reiter, Johannes Wessely, Carlos Baeza, Patricio Peñailillo, Eduardo Ruiz, and Tod Stuessy

**Table S1** Site data of relevés selected from Skottsberg (1953). A-O denotes regions of those relevé sites on the map (Fig. S1).

| Region | Skottsberg Nr | Location | Altitude, m [estim./corr.] |
| --- | --- | --- | --- |
| A | s60 | Plain betw. Quebrada Sanchez & Qu. Larga | 515 |
| B | s75 | Qu. Pasto, table land above | 1100 [900-1100] |
| B | s76 | near 75 | 1100 [900-1100] |
| C | s59 | Qu. Mono | 475 |
| C | s62 | Qu. Mono | 550 |
| D | s61 | Qu. Chozas | 700 |
| D | s74 | Qu. Mono | 720 |
| E | s77 | Corresponecia camp, near stream | 1030 [1000-1050] |
| E | s78 | near 77 | 1020 [1000-1050] |
| F | s80 | Cerro Corresponencia, near summit | 1350 [1250] |
| F | s81 | near 80 | [1250] |
| G | s65 | Qu. Casas S wall, ca. 1km inside | [80] |
| G | s66 | near 66 | [80] |
| G | s67 | Qu. Casas N wall, cliff forest | [80-100] |
| H | s68 | Qu. Casas, at first waterfall | 200 |
| H | s69 | Qu. Casas, at first waterfall | 215 |
| I | s58 | Qu. Blindado | 435 |
| J | s73 | Qu. Vacas N branch | 100 |
| K | s82 | Las Torres, highland nearby | 1350 [1000-1100] |
| K | s83 | Las Torres, under summit | 1350 [1000-1100] |
| K | s84 | Las Torres, summit | 1370 [1000-1100] |
| L | s85 | Los Innocentes, ridge to summit | 1350-1500 [1200-1320] |
| M | s63 | Los Innocentes SE slope | 950 |
| M | s64 | near 63 | [950] |
| N* | s71 | Qu. Angosta, 1 km inside | [200] |
| O | s57 | Qu. Loberia Vieja | 280 |
| O | s72 | Qu. Loberia Vieja | 190 |
| * No recent relevé available from this area, only from the adjacent canyon | | | |

**Table S2** Site data of recent relevés (Greimler & al. 2013) with additional information: Altitude in meters (Alt), aspect (Asp), slope in degrees, cover (C, %) of total vegetation (tot), trees (tree), shrubs (shr), herbs (herb) and bryophytes (bry).

| No. | Location | Alt | Latitude | Longitude | Asp | Slope | C tot | C tree | C shr | C herb | C bry |
| --- | --- | --- | --- | --- | --- | --- | --- | --- | --- | --- | --- |
| 1 | SW Ridge of Tres Torres campsite | 1040 | 33°47'02.9'' | 80°47'17.1'' | NE | 15 | 100 | 0 | 100 | 5 | 5 |
| 2 | W Tres Torres campsite | 1060 | 33°47'05.8'' | 80°47'19.3'' | ENE | 10 | 100 | 5 | 80 | 20 | 5 |
| 3 | Ridge S Q. Vacas towards next valley | 750 | 33°46'53.7'' | 80°46'37.3'' | SE | 40 | 100 | 0 | 90 | 40 | 10 |
| 4 | E C. Innocentes near big rocks | 1180 | 33°47'10.8'' | 80°47'42.7'' | ESE | 20 | 100 | 0 | 100 | 30 | 50 |
| 5 | Ridge N Q. Casas | 730 | 33°45'42.5'' | 80°46'42.9'' | S | 30 | 100 | 0 | 100 | 50 | 10 |
| 6 | Q. de La Cruz | 550 | 33°45'34.6'' | 80°46'19.1'' | SE | 30 | 90 | 5 | 70 | 10 | 5 |
| 7 | above Tres Torres | 980 | 33°46'56.0'' | 80°47'18.9'' | WSW | 40 | 100 | 0 | 95 | 10 | 20 |
| 8 | Valley N Refugio (below pond) | 1020 | 33°45'42.7'' | 80°47'58.1'' | ENE | 0 | 60 | 0 | 10 | 10 | 50 |
| 9 | Cordon Atravesado | 1130 | 33°46'27.9'' | 80°48'15.3'' | S | 40 | 100 | 0 | 100 | 50 | 50 |
| 10 | slope of Q. Guaton - C. Correspondencia | 1220 | 33°45'56.6'' | 80°48'28.5'' | SSW | 30 | 90 | 0 | 85 | 30 | 30 |
| 11 | Avenida de las Cabras | 1120 | 33°45'15.2'' | 80°48'53.6'' | - | 0 | 100 | 0 | 100 | 50 | 10 |
| 12 | Cordon Atravesado | 1170 | 33°46'13.6'' | 80°48'18.7'' | NE | 30 | 95 | 0 | 60 | 30 | 20 |
| 13 | origin of Q. Casas | 1060 | 33°46'06.2'' | 80°48'11.1'' | SE | 30 | 60 | 0 | 40 | 20 | 0 |
| 14 | near origin of Q. Sanchez | 970 | 33°44'50.1'' | 80°48'16.8'' | NE | 5 | 80 | 0 | 75 | 5 | 5 |
| 15 | W above origin of Q. Pasto | 1010 | 33°45'38.3'' | 80°48'04.7'' | NE | 20 | 90 | 0 | 70 | 30 | + |
| 16 | SE Refugio Correspondencia | 1020 | 33°45'42.0'' | 80°47'50.4'' | NE | 35 | 95 | 0 | 85 | 10 | + |
| 17 | SW slope of Cordon Atravesado | 1250 | 33°45'57.6'' | 80°48'25.0'' | SSW | 25 | 65 | 0 | 60 | 10 | 10 |
| 18 | E of Cordon Atravesado | 1210 | 33°45'53.5'' | 80°48'22.5'' | NE | 15 | 85 | 0 | 60 | 20 | 5 |
| 19 | C. Correspondencia, summit area | 1260 | 33°45'48.3'' | 80°48'30.2'' | - | 0 | 60 | 0 | 40 | 10 | 40 |
| 20 | N C. Correspondencia | 1150 | 33°45'33.4'' | 80°48'34.8'' | NE | 35 | 70 | 0 | 30 | 40 | 0 |
| 21 | NW C. Correspondencia | 1180 | 33°45'57.5'' | 80°48'44.2'' | NE | 10 | 60 | 0 | 15 | 45 | 0 |
| 22 | above old Correspondencia campsite | 1080 | 33°45'40.1'' | 80°48'03.5'' | E | 10 | 70 | 0 | 50 | 30 | 5 |
| 23 | Morro del Pasto | 1040 | 33°45'23.2'' | 80°48'02.3'' | NE | 10 | 95 | 0 | 45 | 80 | + |
| 24 | Avenida de las Cabras | 1160 | 33°45'34.0'' | 80°48'44.6'' | NE | 5 | 70 | 0 | 15 | 55 | 0 |
| 25 | Plano de la Mona | 1130 | 33°45'17.9'' | 80°48'34.9'' | E | 10 | 70 | 0 | 30 | 70 | 0 |
| 26 | C. Correspondencia, summit area | 1260 | 33°45'48.3'' | 80°48'30.2'' | - | 0 | 40 | 0 | 30 | 15 | 10 |
| 27 | Ridge N Q. Casas | 510 | 33°45'42.2'' | 80°46'18.2 | NE | 20 | 75 | 0 | 10 | 65 | 0 |
| 28 | C. Correspondencia, near summit | 1250 | 33°45'53.5'' | 80°48'31.0'' | SE | 15 | 95 | 0 | 5 | 95 | + |
| 29 | Ridge N Q. Casas, origin of smaller valleys | 810 | 33°45'45.6'' | 80°46'58.6'' | E | 15 | 85 | 0 | 40 | 50 | 5 |
| 30 | Plano de la Mona | 1070 | 33°45'14.6'' | 80°48'19.6'' | E | 10 | 35 | 0 | 20 | 5 | 0 |
| 31 | NW C. Correspondencia | 1260 | 33°45'49.4'' | 80°48'37.9'' | - | 0 | 40 | 0 | 40 | 5 | 0 |
| 32 | Q. Inocentes, southern valley | 390 | 33°47'02.1'' | 80°45'56.6'' | N | 30 | 100 | 95 | 30 | 70 | 30 |
| 33 | Q. Negra, near origin | 410 | 33°44'18.1'' | 80°47'16.8'' | NE | 20 | 95 | 75 | 50 | 20 | 20 |
| 34 | Plano de Tolten | 670 | 33°44'03.1'' | 80°48'16.4'' | SE | 10 | 100 | 100 | 90 | 5 | 10 |
| 35 | Q. El Tongo | 250 | 33°47'47.9'' | 80°47'57.2'' | SE | 30 | 100 | 20 | 10 | 100 | + |
| 36 | Q. Vacas, northern valley | 130 | 33°46'29.9'' | 80°46'00.8'' | SE | 15 | 100 | 80 | 30 | 50 | 10 |
| 37 | N Q. Pasto (Rebaje La Edionda) | 620 | 33°44'55.9'' | 80°47'13.1'' | S | 30 | 100 | 30 | 60 | 20 | 20 |
| 38 | Q. Larga | 320 | 33°43'42.0'' | 80°47'49.3'' | - | 0 | 95 | 90 | 20 | 40 | + |
| 39 | N Q. Sanchez | 490 | 33°43'46.9'' | 80°47'41.6'' | NE | 5 | 100 | 70 | 25 | 100 | + |
| 40 | above origin of Q. Chozas | 530 | 33°45'23.5'' | 80°46'14.8'' | E | 20 | 60 | 50 | 0 | 50 | 20 |
| 41 | Plano de Sanchez | 400 | 33°43'43.2'' | 80°47'20.5'' | NNW | 30 | 100 | 80 | 20 | 50 | 0 |
| 42 | Origin of Q. Chica | 430 | 33°46'07.1'' | 80°45'54.1'' | NE | 40 | 100 | 45 | 10 | 45 | 0 |
| 43 | Q. Vacas, northern valley | 70 | 33°46'22.7'' | 80°45'45.3'' | S | 40 | 100 | 70 | 0 | 95 | 1 |
| 44 | Ridge of C. Innocentes | 1300 | 33°47'06.0'' | 80°47'59.9'' | S | 10 | 90 | 0 | 0 | 40 | 50 |
| 78 | uppermost slope above Loberia Vieja | 1050 | 33°47'34.3'' | 80°47'09.4 | SW | 40 | 100 | 60 | 40 | 20 | 10 |
| 79 | valley N Refugio (below pond) | 1030 | 33°45'42.7'' | 80°47'58.1'' | - | 0 | 90 | 60 | 30 | 10 | 50 |
| 80 | Valley S Q. Vacas | 750 | 33°46'54.0'' | 80°46'38.0'' | E | 15 | 100 | 0 | 100 | 20 | 30 |
| 81 | Q. Pasto, northern valley | 910 | 33°45'12.1'' | 80°47'53.8'' | all | All | 50 | 0 | 45 | 20 | 20 |
| 82 | Q. Vacas, northern valley | 200 | 33°46'28.6'' | 80°46'38.4'' | N | 90 | 40 | 0 | 0 | 30 | 10 |
| 83 | Q. Vacas, northern valley | 200 | 33°46'28.6'' | 80°46'38.2'' | S | 80 | 70 | 0 | 0 | 50 | 50 |
| 84 | Q. Casas | 180 | 33°46'01.4'' | 80°46'33.0'' | S | 90 | 90 | 0 | 5 | 70 | 80 |
| 86 | Q. Casas | 80 | 33°45'52.3'' | 80°45'59.6'' | SSE | 85 | 60 | 50 | 5 | 20 | 25 |

**Table S3** List of species and their attributes in the 52 relevés selected from Greimler & al. (2013) and in the 27 relevés selected from Skottsberg (1953): fam = family (initials), tgroup = taxonomic group: d dicots, m monocots, f ferns; status: a alien, e endemic, n native non endemic; m(moist) = median moisture preference; m(temp) = median temperature preference; poll = pollination mode; disp = dispersal mode: w wind, z zoochorous, a autochorous.

| **Species** | **Fam** | **tgroup** | **status** | **m(moist)** | **m(temp)** | **poll** | **disp** | **change in occur. frequ.** |
| --- | --- | --- | --- | --- | --- | --- | --- | --- |
| Abrotanella linearifolia | Ast | d | n | very humid | cold | animal | a | -1,78 |
| Acaena argentea | Rosa | d | a | dry | very warm | wind | z | 3,85 |
| Acaena masafuerana | Rosa | d | e | dry | indifferent | animal | z | -6,84 |
| Acaena ovalifolia | Rosa | d | a | dry | warm | animal | z | -1,64 |
| Adiantum chilense | Pterid | f | n | indifferent | warm | humidity | w | 8,40 |
| Agrostis masafuerana | Poac | m | e | very humid | cold | wind | z-w | -3,56 |
| Agrostis stolonifera | Poac | m | a | humid | warm | wind | z-w | -3,70 |
| Aira caryophyllea | Poac | m | a | indifferent | warm | wind | z-w | 23,08 |
| Aira praecox | Poac | m | a | indifferent | warm | wind | z-w | 19,23 |
| Anthoxanthum odoratum | Poac | m | a | dry | warm | wind | z-w | 45,23 |
| Aristotelia chilensis | Eleo | d | a | indifferent | warm | animal | z | 11,54 |
| Asplenium dareoides | Asplen | f | n | very humid | warm | humidity | w | -9,83 |
| Asplenium macrosorum | Asplen | f | e | very humid | indifferent | humidity | w | -7,41 |
| Asplenium obtusatum | Asplen | f | n | very humid | warm | humidity | w | -7,41 |
| Asplenium stellatum | Asplen | f | e | very humid | warm | humidity | w | -5,34 |
| Avena barbata | Poac | m | a | dry | warm | wind | z-w | 3,85 |
| Blechnum chilense | Blechn | f | n | very humid | warm | humidity | w | -0,78 |
| Blechnum cycadifolium | Blechn | f | e | humid | cold | humidity | w | 0,43 |
| Blechnum hastatum | Blechn | f | n | humid | warm | humidity | w | 4,84 |
| Blechnum longicauda | Blechn | f | e | very humid | warm | humidity | w | -27,71 |
| Blechnum mochaenum | Blechn | f | e | very humid | warm | humidity | w | -19,73 |
| Blechnum schottii | Blechn | f | e | very humid | warm | humidity | w | -5,48 |
| Briza minor | Poac | m | a | indifferent | warm | wind | z-w | 3,85 |
| Bromus catharticus | Poac | m | a | dry | warm | wind | z-w | 1,92 |
| Bromus hordeaceus | Poac | m | a | dry | warm | wind | z-w | 1,92 |
| Calystegia tuguriorum | Conv | d | n | indifferent | warm | animal | a | -3,70 |
| Carex banksii | Cyper | m | n | very humid | warm | wind | z-w | -5,48 |
| Carex berteroniana | Cyper | m | e | very humid | warm | wind | z-w | -14,81 |
| Cerastium fontan vulgare | Caryo | d | a | indifferent | warm | animal | a | 2,07 |
| Chaetotropis imberbis | Poac | m | a | dry | very warm | animal | z-w | 1,92 |
| Cirsium vulgare | Ast | d | a | dry | warm | animal | w | 1,92 |
| Coprosma pyrifolia | Rubi | d | e | humid | warm | wind |  | -31,41 |
| Cynosorus echinatus | Ast | m | a | dry | very warm | wind | z-w | 1,92 |
| Cyperus eragrostis | Cyper | m | a | very humid | warm | wind | z-w | 1,92 |
| Cystopteris fragilis | Cystop | f | n | very humid | warm | humidity | w | -12,89 |
| Dendroseris gigantea | Ast | d | e | indifferent | warm | animal | a | -7,41 |
| Dendroseris macrophylla | Ast | d | e | indifferent | warm | animal | a | -14,81 |
| Dendroseris regia | Ast | d | e | very humid | warm | animal | a | -14,81 |
| Dicksonia externa | Dicks | f | e | humid | indifferent | humidity | w | -12,32 |
| Digitalis purpurea | Plant | d | a | indifferent | warm | animal | a | 9,62 |
| Drimys confertifolia | Wint | d | e | very humid | warm | wind | a | -1,35 |
| Empetrum rubrum | Eric | d | n | very humid | cold | wind | z | -3,70 |
| Erigeron fernandezianus | Ast | d | e | indifferent | warm | animal | w | -5,34 |
| Erigeron ingae | Ast | d | e | very humid | cold | animal | w | -7,41 |
| Erigeron luteoviridis | Ast | d | e | very humid | cold | animal | w | -7,41 |
| Erigeron rupicola | Ast | d | e | indifferent | warm | animal | w | -7,41 |
| Erigeron turricola | Ast | d | e | very humid | cold | animal | w | -3,70 |
| Euphrasia formosissima | Orob | d | e | indifferent | warm | animal | w | 2,35 |
| Fagara externa | Ruta | d | e | indifferent | warm | animal | z | -5,20 |
| Galium aparine | Rubi | d | a | indifferent | warm | animal | z | 19,23 |
| Galium masafueranum | Rubi | d | e | very humid | cold | animal | z | -7,41 |
| Gamochaeta alien spec. | Ast | d | a | dry | warm |  |  | 1,92 |
| Gamochaeta fernandeziana | Ast | d | e | dry | warm | animal | w | 11,54 |
| Gavilea insularia | Orch | m | e | humid | cold | animal | z | 3,85 |
| Geranium core-core | Gera | d | a | dry | warm | animal | a | 7,69 |
| Gleichenia lepidota | Gleich | f | e | very humid | cold | humidity | w | 3,85 |
| Gleichenia quadripartita | Gleich | f | n | humid | warm | humidity | w | -5,06 |
| Gleichenia spec. | Gleich | f | e | very humid | cold | humidity | w | -3,70 |
| Gnaphalium spiciformis | Ast | d | a | indifferent | warm | animal |  | -7,41 |
| Gunnera masafuerae | Gunn | d | e | very humid | warm | wind | a | -23,43 |
| Haloragis masafuerana | Halo | d | e | indifferent | warm | wind | a | -9,19 |
| Histiopteris incisa | Denstaedt | f | n | indifferent | warm | humidity | w | -12,61 |
| Holcus lanatus | Poac | m | a | indifferent | warm | wind | z-w | 11,54 |
| Hymenoglossum cruentum | Hymeno | f | n | very humid | warm | humidity | w | -1,78 |
| Hymenophyllum caudiculatum | Hymeno | f | n | very humid | indifferent | humidity | w | -1,64 |
| Hymenophyllum cuneatum | Hymeno | f | n | very humid | warm | humidity | w | -1,07 |
| Hymenophyllum falcland | Hymeno | f | n | very humid | warm | humidity | w | -22,22 |
| Hymenophyllum ferrugin | Hymeno | f | n | very humid | indifferent | humidity | w | -3,70 |
| Hymenophyllum nat. spec. | Hymeno | f | n | very humid | cold | humidity | w | 3,85 |
| Hymenophyllum pectinatum | Hymeno | f | n | very humid | indifferent | humidity | w | -5,34 |
| Hymenophyllum plicatum | Hymeno | f | n | very humid | indifferent | humidity | w | 18,02 |
| Hymenophyllum rugosum | Hymeno | f | e | very humid | indifferent | humidity | w | 10,19 |
| Hymenophyllum tortuosum | Hymeno | f | n | very humid | indifferent | humidity | w | -3,70 |
| Hypericum perforatum | Hyper | d | a | dry | warm | animal | z | 3,85 |
| Hypochaeris radicata | Ast | d | a | indifferent | warm | animal | w | 50,00 |
| Hypolepis poeppigii | Denst | f | n | very humid | warm | humidity | w | -11,11 |
| Lagenophora hariotii | Ast | d | n | humid | cold | animal | a | 6,34 |
| Lapsana communis | Ast | d | a | indifferent | warm | animal | z | 3,85 |
| Libertia chilensis | Irida | m | n | indifferent | warm | animal | a | -10,83 |
| Lobelia alata | Camp | d | n | indifferent | warm | animal | a | -11,11 |
| Lolium perenne | Poac | m | a | indifferent | warm | wind | z-w | 1,92 |
| Lophosoria quadripinnata | Dicks | f | n | humid | indifferent | humidity | w | 26,57 |
| Luzula masafuerana | Junc | m | e | very humid | cold | wind | z-w | 7,98 |
| Lycopodium magellanicum | Lyco | f | n | humid | cold | humidity | w | 21,30 |
| Lycopodium scariosum | Lyco | f | n | very humid | indifferent | humidity | w | -3,70 |
| Megalachne endem. spec. | Poac | m | e | indifferent | warm | wind | z-w | -16,31 |
| Megalastrum inaequalifolium | Dryopt | f | e | very humid | warm | humidity | w | -31,41 |
| Mimulus glabratus | Phryma | d | n | very humid | warm | animal | w | -16,60 |
| Myrceugenia schulzei | Myrt | d | e | indifferent | warm | animal | z | -2,71 |
| Myrteola nummularia | Myrt | d | n | very humid | cold | animal | z | 7,83 |
| Nasella laevissima | Poac | m | n | dry | warm | wind | z-w | 17,31 |
| Nasella neesiana | Poac | m | n | dry | warm | wind | z-w | 7,69 |
| Nertera granadensis | Rubi | d | n | very humid | cold | wind | z | -12,18 |
| Nicotiana cordifolia | Solan | d | e | indifferent | warm | animal | w | -7,41 |
| Oreobolus obtusangulus | Cyper | m | n | very humid | cold | wind | z-w | -3,70 |
| Oxalis alien spec. | Oxal | d | a | dry | warm |  |  | 13,60 |
| Parietaria debilis | Urtic | d | n | indifferent | very warm | wind | w | -9,19 |
| Peperomia berteroana | Piper | d | n | very humid | warm | wind | z | -12,89 |
| Peperomia fernandeziana | Piper | d | n | very humid | warm | wind | z | -5,20 |
| Peperomia skottsbergii | Piper | d | e | very humid | warm | wind | z | -14,81 |
| Pernettya rigida | Eric | d | e | indifferent | warm | wind | z | 33,69 |
| Petroselinum crispum | Apia | d | a | humid | warm | animal | a | -3,70 |
| Piptochaetium bicolor | Poac | m | n | dry | very warm | wind | z-w | 1,92 |
| Plantago lanceolata | Plant | d | a | dry | warm | wind | z | 11,54 |
| Pleopeltis macrocarpa | Polyp | f | n | very humid | warm | humidity | w | -3,56 |
| Polypodium intermedium mas | Polyp | f | e | very humid | warm | humidity | w | -14,81 |
| Polypogon australis | Poac | m | a | indifferent | warm | wind | z-w | -5,48 |
| Polystichum tetragonum | Dryopt | f | e | very humid | warm | humidity | w | -25,78 |
| Potentilla chiloensis | Rosa | d | a | indifferent | warm | animal |  | -3,70 |
| Pteris berteroana | Pterid | f | e | humid | warm | humidity | w | -22,08 |
| Pteris chilensis | Pterid | f | n | humid | warm | humidity | w | 15,53 |
| Pteris semiadnata | Pterid | f | n | very humid | warm | humidity | w | -3,42 |
| Rhaphithamnus venustus | Verb | d | e | very humid | warm | animal | a | -9,19 |
| Robinsonia masafuerae | Ast | d | e | very humid | indifferent | animal | a | -12,89 |
| Rubus geoides | Rosa | d | n | very humid | cold | animal | z | 6,48 |
| Rumex acetosella | Poly | d | a | indifferent | warm | wind | z-w | 56,20 |
| Rumex alien spec. | Poly | d | a | indifferent | warm |  |  | 1,92 |
| Rumex conglomeratus | Poly | d | a | indifferent | warm | wind | a | 3,85 |
| Rumex crispus | Poly | d | a | indifferent | warm | wind | z-w | 1,92 |
| Rumex pulcher | Poly | d | a | indifferent | warm |  | z-w | -3,70 |
| Rumohra berteroana | Dryopt | f | e | indifferent | warm | humidity | w | 29,42 |
| Serpyllopsis caespitosa | Hymeno | f | e | very humid | cold | humidity | w | -11,11 |
| Solanum masafueranum | Solan | d | n | indifferent | warm | animal | z | -3,70 |
| Sonchus alien spec. | Ast | d | a | dry | warm | animal | w | 7,98 |
| Sophora masafuerana | Faba | d | e | dry | warm | animal | a | -9,19 |
| Stellaria media | Caryo | d | a | indifferent | warm | animal | z | -3,56 |
| Thyrsopteris elegans | Thyrs | f | e | very humid | warm | humidity | w | 2,21 |
| Trichomanes exsectum | Hymeno | f | n | very humid | warm | humidity | w | -18,52 |
| Uncinia brevicaulis | Cyper | m | n | very humid | indifferent | wind | z-w | -3,70 |
| Uncinia costata | Cyper | m | e | humid | warm | wind | z-w | -3,70 |
| Uncinia douglasii | Cyper | m | e | very humid | warm | wind | z-w | -14,39 |
| Uncinia tenuis | Cyper | m | n | very humid | warm | wind | z-w | 19,66 |
| Urtica glomeruliflora | Urtic | d | e | indifferent | warm | wind | w | -16,60 |
| Verbena litoralis | Verb | d | a | indifferent | warm | animal |  | -3,70 |
| Vulpia bromoides | Poac | m | a | dry | warm | wind | z-w | 1,92 |
| Wahlenbergia masafuerae | Camp | d | e | indifferent | warm | wind | w | -11,11 |
| Zantedeschia aethiopica | Araceae | m | a | indifferent | warm | animal | z | 1,92 |

**Table S4** Estimated Indicator Values for humidity preferences using a 3-fold scale assigned to each species by six independent experts. Moisture preferences were classified as “dry” (1), “indifferent” (2.0), and “very humid” (3.0).

| **Species** | **S moist** | **T moist** | **N moist** | **B moist** | **P moist** | **L moist** | **Median(moist)** |
| --- | --- | --- | --- | --- | --- | --- | --- |
| Abrotanella linearifolia | 3 | 3 | 3 | 1 | 3 | 3 | **3** |
| Acaena argentea | 1 | 1 | 1 | 1 | 1 | 1 | **1** |
| Acaena masafuerana | 2 | 1 | 3 | 1 | 3 | 1 | **1,5** |
| Acaena ovalifolia | 2 | 1 |  | 1 | 2 | 1 | **1** |
| Adiantum chilense | 2 | 2 | 2 | 2 | 2 | 3 | **2** |
| Agrostis masafuerana | 3 |  | 2 | 2 | 3 | 3 | **3** |
| Agrostis stolonifera | 2 |  |  | 2 | 3 | 3 | **2,5** |
| Aira caryophyllea | 2 |  | 1 | 2 | 2 | 2 | **2** |
| Aira praecox | 2 |  | 1 | 2 | 3 | 1 | **2** |
| Amaranthus deflexus | 2 | 2 |  | 1 | 2 | 1 | **2** |
| Amaryllis belladonna | 2 |  | 1 | 2 | 2 | 1 | **2** |
| Anagallis arvensis | 1 | 2 | 1 | 3 | 2 | 1 | **1,5** |
| Anthoxanthum odoratum | 2 | 1 | 1 | 2 | 1 | 2 | **1,5** |
| Aristotelia chilensis | 2 | 3 | 3 | 2 | 2 | 1 | **2** |
| Asplenium dareoides | 2 | 3 |  | 3 | 2 | 3 | **3** |
| Asplenium macrosorum |  | 3 |  | 3 | 3 | 3 | **3** |
| Asplenium obtusatum | 3 | 3 |  | 3 | 2 | 3 | **3** |
| Asplenium stellatum | 2 | 3 |  | 3 | 3 | 3 | **3** |
| Avena barbata | 1 | 1 | 1 | 1 | 2 | 1 | **1** |
| Bahia ambrosioides | 2 | 2 | 2 | 2 | 2 | 1 | **2** |
| Bidens pilosa | 2 | 2 |  | 1 | 2 | 1 | **2** |
| Blechnum hastatum | 1 | 3 | 3 | 2 | 2 | 3 | **2,5** |
| Blechnum chilense | 2 | 3 | 3 | 3 | 3 | 3 | **3** |
| Blechnum cycadifolium | 2 | 3 | 3 | 1 | 3 | 2 | **2,5** |
| Blechnum longicauda | 3 | 3 | 3 | 3 | 3 | 3 | **3** |
| Blechnum schottii | 2 | 3 | 3 | 3 | 3 | 3 | **3** |
| Blechnum mochaenum | 3 | 3 | 3 | 3 | 2 | 2 | **3** |
| Megalachne berteroana | 2 |  |  | 2 | 2 | 2 | **2** |
| Megalachne masafuerana | 2 |  |  | 2 | 2 | 2 | **2** |
| Brachypodium distachyon | 1 |  |  | 1 |  | 1 | **1** |
| Brassica oleracea | 2 | 2 | 2 | 1 |  | 1 | **2** |
| Briza minor | 1 |  | 2 | 2 | 2 | 1 | **2** |
| Bromus catharticus | 1 |  | 1 | 2 | 2 | 1 | **1** |
| Bromus diandrus | 1 |  |  | 2 | 2 | 1 | **1,5** |
| Bromus hordeaceus | 1 |  | 1 | 2 | 2 | 1 | **1** |
| Bromus lithobius | 1 |  |  | 2 | 2 | 1 | **1,5** |
| Calystegia tuguriorum | 2 |  | 2 | 2 | 2 | 1 | **2** |
| Carex banksii | 2 | 3 | 3 | 3 | 3 | 3 | **3** |
| Carex berteroniana | 3 | 3 |  | 3 | 2 | 3 | **3** |
| Carpobrotus edulis | 1 |  | 2 | 1 | 2 | 1 | **1** |
| Carthamus lanatus | 1 | 2 | 1 |  | 2 | 1 | **1** |
| Cerastium fontanum subsp. vulgare | 2 |  |  | 2 | 2 | 1 | **2** |
| Chaetotropis imberbis | 1 |  |  | 2 | 2 | 1 | **1,5** |
| Chenopodium multifidum | 1 | 2 | 1 | 2 | 2 | 1 | **1,5** |
| Chenopodium murale | 1 | 2 | 1 | 2 | 2 | 1 | **1,5** |
| Cirsium vulgare | 1 | 1 | 1 | 2 | 2 | 1 | **1** |
| Conium maculatum | 2 | 2 | 2 | 2 | 2 | 1 | **2** |
| Convolvulus arvensis | 2 | 2 | 1 | 2 | 2 | 1 | **2** |
| Coprosma pyrifolia | 3 | 3 | 2 | 3 | 2 | 2 | **2,5** |
| Cynosorus echinatus | 1 |  |  | 2 | 2 | 1 | **1,5** |
| Cyperus eragrostis | 2 |  | 3 | 3 | 3 | 3 | **3** |
| Cystopteris fragilis | 3 |  | 3 | 3 | 3 | 2 | **3** |
| Dendroseris macrophylla | 3 |  | 3 | 2 | 2 | 1 | **2** |
| Dicksonia externa | 3 | 3 | 3 | 2 | 2 | 2 | **2,5** |
| Digitalis purpurea | 2 | 2 | 2 | 2 | 2 | 1 | **2** |
| Drimys confertifolia | 3 | 2 | 3 | 3 | 3 | 2 | **3** |
| Megalastrum inaequalifolium | 3 | 3 |  | 3 | 3 | 1 | **3** |
| Eleusine tristachya |  |  |  | 2 |  |  | **2** |
| Empetrum rubrum | 3 | 2 | 3 | 1 | 3 | 3 | **3** |
| Erigeron fernandezianus | 2 | 2 | 2 | 3 | 2 | 1 | **2** |
| Erigeron ingae | 3 | 2 | 3 | 3 | 3 | 3 | **3** |
| Erigeron luteoviridis | 3 | 2 | 2 | 3 | 3 | 3 | **3** |
| Erigeron rupicola | 2 | 3 | 2 | 3 | 2 | 1 | **2** |
| Erigeron turricola | 3 | 2 | 2 | 3 | 3 | 3 | **3** |
| Eryngium sarcophyllum |  | 3 |  | 2 |  | 1 | **2** |
| Euphorbia peplus | 1 | 2 | 2 | 2 | 2 | 1 | **2** |
| Euphrasia formosissima | 2 | 3 | 2 | 2 | 2 | 3 | **2** |
| Fagara externa | 2 | 1 | 3 | 3 | 2 | 1 | **2** |
| Potentilla chiloensis |  | 2 | 3 | 2 | 2 |  | **2** |
| Galinsoga parviflora | 1 | 2 | 2 | 2 | 2 | 1 | **2** |
| Galium aparine | 2 | 2 | 1 | 2 | 2 | 1 | **2** |
| Galium masafueranum | 3 |  | 1 | 2 | 3 | 3 | **3** |
| Gamochaeta coarctata | 2 | 1 |  | 1 |  |  | **1** |
| Gamochaeta fernandeziana |  | 1 |  | 1 |  | 1 | **1** |
| Gamochaeta species |  | 1 | 1 |  |  |  | **1** |
| Gamochaeta stachydifolia |  | 1 |  | 1 | 2 |  | **1** |
| Gavilea insularia | 3 |  | 2 | 2 |  | 3 | **2,5** |
| Geranium core-core | 1 | 1 | 2 | 1 | 2 | 1 | **1** |
| Gleichenia quadripartita | 2 | 2 | 3 | 3 | 3 | 2 | **2,5** |
| Gleichenia lepidota | 3 | 2 | 3 | 3 | 3 | 3 | **3** |
| Gnaphalium spiciformis | 2 | 2 |  | 1 | 2 | 2 | **2** |
| Gunnera masafuerae | 3 | 3 | 3 | 3 | 3 | 3 | **3** |
| Haloragis masafuerana | 2 | 1 | 3 | 3 | 2 | 1 | **2** |
| Dendroseris gigantea |  | 2 | 3 | 2 | 2 | 1 | **2** |
| Histiopteris incisa | 2 |  | 2 | 3 | 2 | 1 | **2** |
| Holcus lanatus | 2 |  |  | 2 | 2 | 1 | **2** |
| Hordeum murinum | 1 |  | 2 | 1 | 2 | 1 | **1** |
| Hymenoglossum cruentum | 2 | 3 |  | 3 | 3 | 3 | **3** |
| Hymenophyllum caudiculatum | 2 | 3 | 3 | 3 | 3 | 3 | **3** |
| Hymenophyllum cuneatum | 2 | 3 | 3 | 3 | 3 | 3 | **3** |
| Hymenophyllum falclandicum | 2 | 3 | 3 | 3 | 3 | 3 | **3** |
| Hymenophyllum ferrugineum | 2 | 3 | 3 | 3 | 3 | 3 | **3** |
| Hymenophyllum pectinatum | 2 | 3 | 3 | 3 | 3 | 3 | **3** |
| Hymenophyllum plicatum | 2 | 3 | 3 | 3 | 3 | 3 | **3** |
| Hymenophyllum rugosum | 2 | 3 | 3 | 3 | 3 | 3 | **3** |
| Hymenophyllum tortuosum | 2 | 3 | 3 | 3 | 3 | 3 | **3** |
| Hymenophyllum species UNCLEAR |  | 3 | 3 | 3 |  |  | **3** |
| Hypericum perforatum | 2 | 1 | 3 | 1 | 2 | 1 | **1,5** |
| Hypochaeris radicata | 2 | 2 | 1 | 1 | 2 | 2 | **2** |
| Hypolepis poeppigii |  |  | 3 | 3 | 3 | 2 | **3** |
| Juncus bufonius | 2 | 3 | 2 | 3 | 3 | 3 | **3** |
| Juncus capillaceus | 2 | 3 | 2 | 3 | 3 | 2 | **2,5** |
| Juncus procerus | 2 | 3 | 2 | 3 | 3 | 1 | **2,5** |
| Lagenophora hariotii | 2 | 1 | 3 | 2 | 3 | 3 | **2,5** |
| Lapsana communis | 2 | 2 |  | 2 | 3 | 1 | **2** |
| Libertia chilensis | 2 | 2 | 3 | 3 | 2 | 1 | **2** |
| Lobelia alata | 1 | 2 |  | 3 | 3 | 1 | **2** |
| Lolium perenne | 2 |  | 1 | 2 | 2 | 2 | **2** |
| Lophosoria quadripinnata | 2 | 2 | 3 | 3 | 2 | 3 | **2,5** |
| Luzula masafuerana | 3 | 3 | 2 | 3 | 3 | 3 | **3** |
| Lycopodium magellanicum | 2 | 2 | 2 | 3 | 3 | 3 | **2,5** |
| Lycopodium scariosum |  |  |  | 3 | 3 |  | **3** |
| Marrubium vulgare | 1 |  | 2 | 3 | 3 | 3 | **3** |
| Mimulus glabratus | 2 |  | 3 | 3 | 3 | 3 | **3** |
| Modiola caroliniana |  |  |  | 2 | 3 | 1 | **2** |
| Myrceugenia schulzei | 2 | 2 | 2 | 3 | 2 | 2 | **2** |
| Myrteola nummularia | 3 |  | 3 | 2 | 3 | 3 | **3** |
| Nassella laevissima | 1 |  | 1 | 2 | 1 | 2 | **1** |
| Nassella neesiana | 1 |  | 1 | 2 | 1 | 2 | **1** |
| Nertera granadensis | 3 | 2 | 3 | 3 | 3 | 3 | **3** |
| Nicotiana cordifolia |  |  |  | 2 | 2 | 1 | **2** |
| Oenothera rosea | 1 | 2 | 2 | 1 | 3 | 1 | **1,5** |
| Oreobolus obtusangulus | 3 |  | 3 |  | 3 | 3 | **3** |
| Oxalis corniculata | 2 | 1 |  | 2 | 2 | 1 | **2** |
| Oxalis micrantha | 2 | 1 |  | 2 | 2 | 1 | **2** |
| Oxalis species |  | 1 | 2 |  |  |  | **1,5** |
| Parietaria debilis | 2 |  | 1 |  | 2 | 3 | **2** |
| Paronychia franciscana | 1 |  |  |  | 2 |  | **1,5** |
| Paronychia species | 1 |  |  |  |  |  | **1** |
| Notholaena chilensis |  |  | 1 | 2 | 1 | 1 | **1** |
| Peperomia berteroana | 3 | 1 | 3 | 3 | 3 | 3 | **3** |
| Peperomia fernandeziana | 3 | 3 | 3 | 3 | 3 | 3 | **3** |
| Peperomia skottsbergii | 3 | 3 | 3 | 3 | 3 | 3 | **3** |
| Pernettya rigida | 2 | 2 | 3 | 2 | 2 | 2 | **2** |
| Petroselinum crispum | 2 |  |  | 2 | 3 | 3 | **2,5** |
| Dendroseris regia | 3 |  | 3 | 2 | 3 |  | **3** |
| Physalis peruviana |  | 2 | 2 | 2 | 3 | 1 | **2** |
| Piptochaetium bicolor | 2 |  |  | 2 | 1 | 1 | **1,5** |
| Plantago lanceolata | 2 | 1 | 1 | 2 | 2 | 1 | **1,5** |
| Polycarpon tetraphyllum | 1 |  |  |  | 2 |  | **1,5** |
| Polygonum aviculare | 2 |  | 1 | 2 | 3 | 2 | **2** |
| Polypodium intermedium ssp masa | 2 |  | 3 | 3 | 3 | 1 | **3** |
| Pleopeltis macrocarpa | 3 |  | 3 | 3 | 3 |  | **3** |
| Polypogon australis | 1 |  |  | 2 | 3 |  | **2** |
| Rumohra berteroana | 1 |  | 3 | 3 | 2 | 2 | **2** |
| Polystichum tetragonum | 2 |  |  | 3 | 3 | 3 | **3** |
| Pteris berteroana | 2 |  |  | 3 | 3 | 2 | **2,5** |
| Pteris chilensis | 2 | 2 | 3 | 3 | 3 | 2 | **2,5** |
| Pteris semiadnata | 2 |  | 3 | 3 | 3 | 2 | **3** |
| Rhaphithamnus venustus | 3 | 3 | 3 | 3 | 3 | 1 | **3** |
| Robinsonia masafuerae | 3 | 2 | 3 | 3 | 3 | 3 | **3** |
| Rubus geoides | 3 | 2 | 3 | 2 | 3 | 3 | **3** |
| Rumex acetosella | 1 | 2 | 2 | 1 | 2 | 2 | **2** |
| Rumex conglomeratus | 2 | 2 | 2 | 1 | 2 | 1 | **2** |
| Rumex crispus | 2 | 2 | 2 | 1 | 2 | 1 | **2** |
| Rumex pulcher | 2 | 2 | 2 | 1 | 2 | 1 | **2** |
| Rumex species |  | 2 | 2 | 1 |  |  | **2** |
| Ruta chalepensis | 1 | 2 | 2 | 2 | 2 | 1 | **2** |
| Sarcocornia fruticosa | 1 | 1 | 3 | 3 | 2 |  | **2** |
| Scirpus nodosus | 2 |  |  | 3 | 2 | 3 | **2,5** |
| Senecio vulgaris | 2 | 2 | 1 | 2 | 2 | 1 | **2** |
| Setaria parviflora | 1 |  |  | 2 | 2 |  | **2** |
| Serpyllopsis caespitosa |  |  | 3 | 3 | 3 | 3 | **3** |
| Silene gallica | 1 |  | 1 | 2 | 2 | 2 | **2** |
| Silybum marianum | 1 | 1 | 1 | 2 | 2 | 1 | **1** |
| Solanum masafueranum |  | 2 |  | 2 | 3 | 1 | **2** |
| Sonchus asper/oleraceus | 1 | 2 | 1 |  | 2 | 1 | **1** |
| Sophora masafuerana | 2 | 1 | 1 | 3 | 3 | 1 | **1,5** |
| Spergularia confertiflora | 2 | 3 | 1 | 2 | 2 | 1 | **2** |
| Spergularia masafuerana | 2 | 3 | 1 | 2 | 1 | 1 | **1,5** |
| Stellaria media | 2 | 2 | 2 | 2 | 2 | 1 | **2** |
| Taraxacum officinale | 2 | 2 | 1 | 2 | 2 | 2 | **2** |
| Tetragonia tetragonioides | 1 |  | 1 | 2 | 3 |  | **1,5** |
| Thyrsopteris elegans | 3 | 3 | 3 | 3 | 3 | 2 | **3** |
| Trichomanes exsectum | 3 |  |  | 3 | 3 | 3 | **3** |
| Uncinia brevicaulis |  | 3 |  | 3 | 3 | 3 | **3** |
| Uncinia costata |  | 3 |  | 3 | 2 | 1 | **2,5** |
| Uncinia douglasii | 3 | 3 | 3 | 3 | 2 | 1 | **3** |
| Uncinia tenuis | 3 | 3 |  | 3 | 3 | 1 | **3** |
| Urtica glomeruliflora |  |  |  | 3 | 2 | 1 | **2** |
| Verbascum thapsus | 2 | 2 | 1 | 2 | 1 | 1 | **1,5** |
| Verbascum virgatum | 2 | 2 | 1 | 2 | 2 | 1 | **2** |
| Verbena litoralis | 1 | 2 | 2 | 2 | 2 | 1 | **2** |
| Vicia hirsuta | 2 |  | 1 | 2 | 2 | 1 | **2** |
| Vulpia bromoides | 1 |  | 1 | 2 | 1 | 2 | **1** |
| Wahlenbergia masafuerae | 2 | 1 | 1 | 3 | 2 | 3 | **2** |
| Zantedeschia aethiopica | 2 | 2 | 2 | 3 | 2 | 1 | **2** |

**Table S5** Estimated Indicator Values for temperature preferences using a 3-fold scale assigned to each species by six independent experts. Temperature preferences were classified as “warm” (1), “indifferent” (2.0), and “cold” (3.0).

| **Median(moist)** | **S temp** | **T temp** | **N temp** | **B temp** | **P temp** | **L temp** | **Median(moist)** |
| --- | --- | --- | --- | --- | --- | --- | --- |
| Abrotanella linearifolia | 3 | 3 | 3 | 3 | 3 | 1 | **3** |
| Acaena argentea | 1 | 2 | 2 | 1 | 2 | 1 | **1,5** |
| Acaena masafuerana | 3 | 2 | 2 | 3 | 3 | 1 | **2,5** |
| Acaena ovalifolia | 2 | 2 |  | 3 | 2 | 1 | **2** |
| Adiantum chilense | 2 | 2 | 2 | 2 | 2 | 2 | **2** |
| Agrostis masafuerana | 3 |  | 2 | 2 | 3 | 3 | **3** |
| Agrostis stolonifera | 2 |  |  | 2 | 2 | 2 | **2** |
| Aira caryophyllea | 1 |  | 2 | 2 | 2 | 2 | **2** |
| Aira praecox | 2 |  | 2 | 2 | 3 | 2 | **2** |
| Amaranthus deflexus | 1 | 2 |  | 1 | 2 | 1 | **1** |
| Amaryllis belladonna | 1 |  | 2 | 2 | 2 | 1 | **2** |
| Anagallis arvensis | 1 | 2 | 2 | 2 | 2 | 1 | **2** |
| Anthoxanthum odoratum | 2 | 2 | 2 | 2 | 3 | 2 | **2** |
| Aristotelia chilensis | 2 | 2 | 2 | 2 | 2 | 2 | **2** |
| Asplenium dareoides | 2 | 2 |  | 2 | 2 | 3 | **2** |
| Asplenium macrosorum |  | 3 |  | 2 | 1 | 3 | **2,5** |
| Asplenium obtusatum | 1 | 2 |  | 2 | 2 | 2 | **2** |
| Asplenium stellatum | 2 | 2 |  | 2 | 2 | 3 | **2** |
| Avena barbata | 1 | 2 | 2 | 2 | 2 | 1 | **2** |
| Bahia ambrosioides | 1 | 2 | 2 | 2 | 2 | 1 | **2** |
| Bidens pilosa | 1 | 2 |  | 2 | 2 | 1 | **2** |
| Blechnum hastatum | 2 | 2 | 2 | 2 | 2 | 1 | **2** |
| Blechnum chilense | 2 | 2 | 2 | 2 | 2 | 1 | **2** |
| Blechnum cycadifolium | 3 | 3 | 3 | 2 | 2 | 3 | **3** |
| Blechnum longicauda | 2 | 2 | 2 | 2 | 2 | 1 | **2** |
| Blechnum schottii | 2 | 2 | 2 | 2 | 2 | 1 | **2** |
| Blechnum mochaenum | 2 | 2 | 3 | 2 | 2 |  | **2** |
| Megalachne berteroana | 2 |  |  | 2 | 2 | 3 | **2** |
| Megalachne masafuerana | 2 |  |  | 2 | 2 | 3 | **2** |
| Brachypodium distachyon | 1 |  |  | 2 |  | 1 | **1** |
| Brassica oleracea | 1 | 2 | 2 | 2 |  | 1 | **2** |
| Briza minor | 1 |  | 2 | 2 | 2 | 1 | **2** |
| Bromus catharticus | 1 |  | 2 | 2 | 2 | 1 | **2** |
| Bromus diandrus | 1 |  |  | 2 | 2 | 1 | **1,5** |
| Bromus hordeaceus | 1 |  | 2 | 2 | 2 | 1 | **2** |
| Bromus lithobius | 1 |  |  | 2 | 2 | 1 | **1,5** |
| Calystegia tuguriorum | 2 |  | 2 | 2 | 2 | 1 | **2** |
| Carex banksii | 2 | 2 | 2 | 2 | 2 | 1 | **2** |
| Carex berteroniana | 2 | 2 |  | 2 | 2 | 1 | **2** |
| Carpobrotus edulis | 1 |  | 2 | 2 | 2 | 1 | **2** |
| Carthamus lanatus | 1 | 2 | 2 |  | 2 | 1 | **2** |
| Cerastium fontanum subsp. vulgare | 2 |  |  | 2 | 2 | 1 | **2** |
| Chaetotropis imberbis | 1 |  |  | 2 | 1 | 1 | **1** |
| Chenopodium multifidum | 1 | 2 | 2 | 2 | 2 | 1 | **2** |
| Chenopodium murale | 1 | 2 | 2 | 2 | 2 | 1 | **2** |
| Cirsium vulgare | 2 | 2 | 1 | 2 | 2 | 1 | **2** |
| Conium maculatum | 2 | 2 | 2 | 2 | 2 | 1 | **2** |
| Convolvulus arvensis | 2 | 2 | 2 | 2 | 2 | 1 | **2** |
| Coprosma pyrifolia | 2 | 2 | 2 | 2 | 2 | 2 | **2** |
| Cynosorus echinatus | 1 |  |  | 2 | 2 | 1 | **1,5** |
| Cyperus eragrostis | 2 |  | 2 | 2 | 2 | 1 | **2** |
| Cystopteris fragilis | 2 |  | 2 | 2 | 2 | 1 | **2** |
| Dendroseris macrophylla | 2 |  | 3 | 2 | 2 | 1 | **2** |
| Dicksonia externa | 3 | 2 | 3 | 2 | 2 | 3 | **2,5** |
| Digitalis purpurea | 2 | 2 | 2 | 2 | 2 | 1 | **2** |
| Drimys confertifolia | 3 | 2 | 3 | 2 | 2 | 2 | **2** |
| Megalastrum inaequalifolium | 2 | 2 |  | 2 | 2 | 1 | **2** |
| Eleusine tristachya |  |  |  | 2 |  |  | **2** |
| Empetrum rubrum | 3 | 3 | 3 | 2 | 3 | 3 | **3** |
| Erigeron fernandezianus | 2 | 2 | 2 | 2 | 2 | 3 | **2** |
| Erigeron ingae | 3 | 3 | 2 | 2 | 3 | 3 | **3** |
| Erigeron luteoviridis | 3 | 3 | 2 | 2 | 3 | 3 | **3** |
| Erigeron rupicola | 2 | 2 | 2 | 2 | 2 | 1 | **2** |
| Erigeron turricola | 3 | 3 | 2 | 2 | 3 | 3 | **3** |
| Eryngium sarcophyllum |  | 1 |  | 2 |  | 1 | **1** |
| Euphorbia peplus | 1 | 2 | 2 | 2 | 2 | 1 | **2** |
| Euphrasia formosissima | 2 | 2 | 2 | 2 | 2 | 3 | **2** |
| Fagara externa | 2 | 1 | 2 | 2 | 2 | 1 | **2** |
| Potentilla chiloensis |  | 2 | 2 | 2 | 2 |  | **2** |
| Galinsoga parviflora | 2 | 2 | 2 | 2 | 2 | 1 | **2** |
| Galium aparine | 2 | 2 | 2 | 2 | 2 | 1 | **2** |
| Galium masafueranum | 3 |  | 2 | 2 | 3 | 3 | **3** |
| Gamochaeta coarctata | 2 | 2 |  | 2 |  |  | **2** |
| Gamochaeta fernandeziana |  | 2 |  | 2 |  | 1 | **2** |
| Gamochaeta species |  | 2 | 2 |  |  |  | **2** |
| Gamochaeta stachydifolia |  | 2 |  | 2 | 2 |  | **2** |
| Gavilea insularia | 3 |  | 3 | 2 |  | 3 | **3** |
| Geranium core-core | 2 | 2 | 2 | 2 | 2 | 1 | **2** |
| Gleichenia quadripartita | 2 | 2 | 3 | 2 | 3 | 2 | **2** |
| Gleichenia lepidota | 3 | 2 | 3 | 2 | 3 |  | **3** |
| Gnaphalium spiciformis | 2 | 2 |  | 2 | 2 | 2 | **2** |
| Gunnera masafuerae | 2 | 2 | 2 | 2 | 2 | 2 | **2** |
| Haloragis masafuerana | 2 | 2 | 2 | 2 | 2 | 1 | **2** |
| Dendroseris gigantea |  | 2 | 2 | 2 | 2 | 1 | **2** |
| Histiopteris incisa | 2 |  | 2 | 2 | 2 | 2 | **2** |
| Holcus lanatus | 2 |  |  | 2 | 2 | 1 | **2** |
| Hordeum murinum | 2 |  | 2 | 2 | 2 | 1 | **2** |
| Hymenoglossum cruentum | 2 | 3 |  | 2 | 2 | 3 | **2** |
| Hymenophyllum caudiculatum | 2 | 3 | 3 | 2 | 2 | 3 | **2,5** |
| Hymenophyllum cuneatum | 2 | 3 | 3 | 2 | 2 | 2 | **2** |
| Hymenophyllum falclandicum | 2 | 3 | 3 | 2 | 2 | 2 | **2** |
| Hymenophyllum ferrugineum | 2 | 3 | 3 | 2 | 2 | 3 | **2,5** |
| Hymenophyllum pectinatum | 2 | 3 | 3 | 2 | 2 | 3 | **2,5** |
| Hymenophyllum plicatum | 2 | 3 | 3 | 2 | 2 | 3 | **2,5** |
| Hymenophyllum rugosum | 2 | 3 | 3 | 2 | 2 | 3 | **2,5** |
| Hymenophyllum tortuosum | 2 | 3 | 3 | 2 | 2 | 3 | **2,5** |
| Hymenophyllum species UNCLEAR |  | 3 | 3 | 2 |  |  | **3** |
| Hypericum perforatum | 1 | 2 | 2 | 2 | 2 | 1 | **2** |
| Hypochaeris radicata | 2 | 2 | 2 | 2 | 2 | 2 | **2** |
| Hypolepis poeppigii |  |  | 2 | 2 | 2 | 2 | **2** |
| Juncus bufonius | 1 | 2 | 2 | 2 | 2 | 1 | **2** |
| Juncus capillaceus | 2 | 2 | 2 | 2 | 2 | 2 | **2** |
| Juncus procerus | 2 | 2 | 2 | 2 | 2 | 1 | **2** |
| Lagenophora hariotii | 2 | 3 | 3 | 2 | 3 | 3 | **3** |
| Lapsana communis | 2 | 2 |  | 2 | 2 | 1 | **2** |
| Libertia chilensis | 2 | 2 | 2 | 2 | 2 |  | **2** |
| Lobelia alata | 1 | 2 |  | 2 | 2 | 1 | **2** |
| Lolium perenne | 2 |  | 2 | 2 | 2 | 2 | **2** |
| Lophosoria quadripinnata | 3 | 3 | 2 | 2 | 2 | 3 | **2,5** |
| Luzula masafuerana | 3 | 2 | 3 | 2 | 3 | 3 | **3** |
| Lycopodium magellanicum | 3 | 2 | 3 | 2 | 3 | 3 | **3** |
| Lycopodium scariosum |  |  |  | 2 | 3 |  | **2,5** |
| Marrubium vulgare | 1 |  | 2 | 2 | 2 | 2 | **2** |
| Mimulus glabratus | 1 |  | 2 | 2 | 2 | 3 | **2** |
| Modiola caroliniana |  |  |  | 2 | 2 | 1 | **2** |
| Myrceugenia schulzei | 2 | 1 | 2 | 2 | 2 | 2 | **2** |
| Myrteola nummularia | 3 |  | 3 | 2 | 3 | 3 | **3** |
| Nassella laevissima | 2 |  | 2 | 2 | 1 | 2 | **2** |
| Nassella neesiana | 1 |  | 2 | 2 | 1 | 2 | **2** |
| Nertera granadensis | 3 | 3 | 3 | 2 | 2 | 3 | **3** |
| Nicotiana cordifolia |  |  |  | 2 | 2 | 1 | **2** |
| Oenothera rosea | 1 | 2 | 2 | 2 | 2 | 1 | **2** |
| Oreobolus obtusangulus | 3 |  | 3 |  | 3 | 3 | **3** |
| Oxalis corniculata |  | 2 |  | 2 | 2 | 1 | **2** |
| Oxalis micrantha | 2 | 2 |  | 2 | 2 | 1 | **2** |
| Oxalis species |  | 2 | 2 |  |  |  | **2** |
| Parietaria debilis | 1 |  | 2 |  | 2 | 1 | **1,5** |
| Paronychia franciscana | 1 |  |  |  | 2 |  | **1,5** |
| Paronychia species | 1 |  |  |  |  |  | **1** |
| Notholaena chilensis |  |  | 2 | 2 | 1 | 1 | **1,5** |
| Peperomia berteroana | 2 | 2 | 3 | 2 | 2 | 2 | **2** |
| Peperomia fernandeziana | 2 | 2 | 3 | 2 | 2 | 2 | **2** |
| Peperomia skottsbergii | 2 | 1 | 3 | 2 | 2 | 2 | **2** |
| Pernettya rigida | 2 | 2 | 2 | 2 | 2 | 2 | **2** |
| Petroselinum crispum | 2 |  |  | 2 | 2 | 1 | **2** |
| Dendroseris regia | 2 |  | 2 | 2 | 2 |  | **2** |
| Physalis peruviana |  | 2 | 2 | 2 | 2 | 1 | **2** |
| Piptochaetium bicolor | 2 |  |  | 2 | 1 | 1 | **1,5** |
| Plantago lanceolata | 2 | 2 | 2 | 2 | 2 | 1 | **2** |
| Polycarpon tetraphyllum | 1 |  |  |  | 2 |  | **1,5** |
| Polygonum aviculare | 2 |  | 2 | 2 | 2 | 2 | **2** |
| Polypodium intermedium ssp masa | 2 |  | 3 | 2 | 2 | 1 | **2** |
| Pleopeltis macrocarpa | 2 |  | 3 | 2 | 2 |  | **2** |
| Polypogon australis | 1 |  |  | 2 | 2 |  | **2** |
| Rumohra berteroana | 2 |  | 3 | 2 | 2 | 2 | **2** |
| Polystichum tetragonum | 2 |  |  | 2 | 2 | 3 | **2** |
| Pteris berteroana | 2 |  |  | 2 | 2 | 1 | **2** |
| Pteris chilensis | 2 | 2 | 2 | 2 | 2 | 2 | **2** |
| Pteris semiadnata | 2 |  | 2 | 2 | 2 | 1 | **2** |
| Rhaphithamnus venustus | 2 | 2 | 3 | 2 | 2 | 3 | **2** |
| Robinsonia masafuerae | 3 | 2 | 3 | 2 | 2 | 3 | **2,5** |
| Rubus geoides | 3 | 3 | 2 | 2 | 3 | 3 | **3** |
| Rumex acetosella | 2 | 2 | 2 | 2 | 2 | 2 | **2** |
| Rumex conglomeratus | 2 | 2 | 2 | 2 | 2 | 1 | **2** |
| Rumex crispus | 2 | 2 | 2 | 2 | 2 | 1 | **2** |
| Rumex pulcher | 2 | 2 | 2 | 2 | 2 | 1 | **2** |
| Rumex species |  | 2 | 2 | 2 |  |  | **2** |
| Ruta chalepensis | 1 | 2 | 2 | 2 | 2 | 1 | **2** |
| Sarcocornia fruticosa | 1 | 3 | 2 | 2 | 2 |  | **2** |
| Scirpus nodosus | 2 |  |  | 2 | 2 | 1 | **2** |
| Senecio vulgaris | 2 | 2 | 2 | 2 | 2 | 1 | **2** |
| Setaria parviflora | 1 |  |  | 2 | 2 |  | **2** |
| Serpyllopsis caespitosa |  |  | 3 | 2 | 3 | 3 | **3** |
| Silene gallica | 1 |  | 2 | 2 | 2 | 1 | **2** |
| Silybum marianum | 1 | 2 | 2 | 2 | 2 | 1 | **2** |
| Solanum masafueranum |  | 2 |  | 2 | 2 | 1 | **2** |
| Sonchus asper/oleraceus | 2 | 2 | 2 |  | 2 | 1 | **2** |
| Sophora masafuerana | 2 | 2 | 2 | 2 | 2 | 1 | **2** |
| Spergularia confertiflora | 1 | 1 | 2 | 2 | 2 | 1 | **1,5** |
| Spergularia masafuerana | 1 | 1 | 2 | 2 | 2 | 1 | **1,5** |
| Stellaria media | 2 | 2 | 2 | 2 | 2 | 1 | **2** |
| Taraxacum officinale | 2 | 2 | 2 | 2 | 2 | 2 | **2** |
| Tetragonia tetragonioides | 1 |  | 1 | 2 | 2 |  | **1,5** |
| Thyrsopteris elegans | 2 | 3 | 3 | 2 | 2 | 2 | **2** |
| Trichomanes exsectum | 2 |  |  | 2 | 2 | 2 | **2** |
| Uncinia brevicaulis |  | 2 |  | 2 | 3 | 3 | **2,5** |
| Uncinia costata |  | 2 |  | 2 | 2 | 1 | **2** |
| Uncinia douglasii | 2 | 2 | 2 | 2 | 2 | 1 | **2** |
| Uncinia tenuis | 3 | 2 |  | 2 | 3 | 1 | **2** |
| Urtica glomeruliflora |  |  |  | 2 | 2 | 1 | **2** |
| Verbascum thapsus | 2 | 2 | 2 | 2 | 2 | 1 | **2** |
| Verbascum virgatum | 1 | 2 | 2 | 2 | 2 | 1 | **2** |
| Verbena litoralis | 1 | 2 | 2 | 2 | 2 | 1 | **2** |
| Vicia hirsuta | 2 |  | 2 | 2 | 2 | 1 | **2** |
| Vulpia bromoides | 1 |  | 2 | 2 | 2 | 2 | **2** |
| Wahlenbergia masafuerae | 2 | 2 | 2 | 2 | 2 | 3 | **2** |
| Zantedeschia aethiopica | 2 | 2 | 2 | 2 | 2 | 1 | **2** |

**Note:** Tables S4 and S5 contain the complete list of taxa including those occurring only in the grasslands. Grasslands were excluded from our analysis (see methods section).

**Table S6** Survey effort and number of recorded native non-endemic, endemic and alien plant species in 1917 and 2011. The percentages the respective groups are contributing to the respective floras in 1917 and 2011 are provided in brackets.

| Considered | Year | Sampled | Number of species | | | |
| --- | --- | --- | --- | --- | --- | --- |
| Taxa |  | plots | Total | Native non-endemics | Endemics | Aliens |
| All | 1917 | 27 | 103 | 42 (40.8 %) | 47 (45.6 %) | 14 (13.6 %) |
|  | 2011 | 52 | 101 | 33 (32.7%) | 33 (32.7 %) | 35 (34.6 %) |
| Monocots | 1917 | 27 | 14 | 5 (35.7 %) | 6 (42.9 %) | 3 (21.4 %) |
|  | 2011 | 52 | 26 | 6 (23.1 %) | 5 (19.2 %) | 15 (57.7 %) |
| Dicots | 1917 | 27 | 49 | 13 (26.5 %) | 25 (51.0 %) | 11 (22.4 %) |
|  | 2011 | 52 | 44 | 9 (20.4 %) | 15 (34.1 %) | 20 (45.5 %) |
| Ferns | 1917 | 27 | 40 | 24 (60.0 %) | 16 (40.0 %) | 0 (0.0 %) |
|  | 2011 | 52 | 31 | 18 (58.1 %) | 13 (41.9 %) | 0 (0.0 %) |


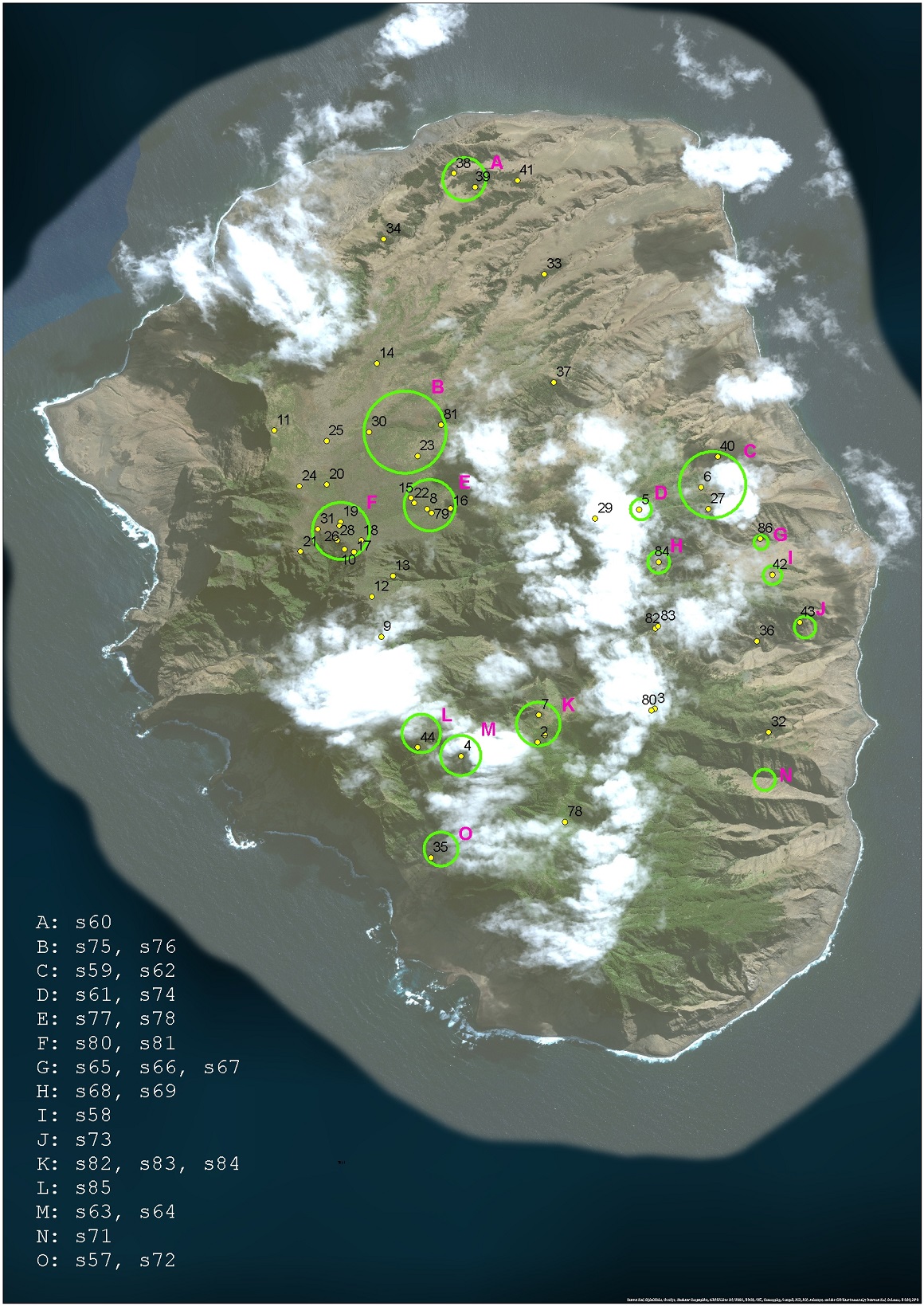


**Figure S1** The island Alejandro Selkirk with the historical sampling sites of 1917 indicated by the green circles A-O with each circle containing between 1 and 3 relevés of Skottsberg (see bottom left). The recent relevés of 2011 are indicated by the yellow dots inside and outside these circles.

**Figure S2** Annual precipitation on Robinson Crusoe island (A) and mean annual temperature on this island and Valparaiso on the Chilean coast (B, C).
